# Unscheduled hospital contacts after inpatient discharge: A national observational study of COPD and heart failure patients in England

**DOI:** 10.1371/journal.pone.0218128

**Published:** 2019-06-13

**Authors:** Kate Honeyford, Derek Bell, Faiza Chowdhury, Jennifer Quint, Paul Aylin, Alex Bottle

**Affiliations:** 1 Digital Health Unit, Department of Primary Care and Public Health, Imperial College, London, United Kingdom; 2 School of Public Health, Imperial College London, Chelsea and Westminster Campus, London, United Kingdom; 3 NIHR CLAHRC for North West London, Imperial College London, Chelsea & Westminster Campus, London, United Kingdom; 4 National Heart and Lung Institute, Imperial College London, Royal Brompton Campus, London, United Kingdom; 5 Dr Foster Unit, Department of Primary care and Public Health, Imperial College, London, United Kingdom; University of Malta Faculty of Health Sciences, MALTA

## Abstract

**Introduction:**

Readmissions are a recognised challenge for providers of healthcare and incur financial penalties in a growing number of countries. However, the scale of unscheduled hospital contacts including attendances at emergency departments that do not result in admission is not well known. In addition, little is known about the route to readmission for patients recently discharged from an emergency hospital stay.

**Methods:**

This is an observational study of national hospital administration data for England. In this retrospective cohort study, we tracked patients for 30 days after discharge from an emergency admission for heart failure (HF) or chronic obstructive pulmonary disorder (COPD).

**Results:**

The majority of patients (COPD:79%; HF:75%) had no unscheduled contact with secondary health care within 30 days of discharge. Of those who did have unscheduled contact, the most common first unscheduled contact was emergency department (ED) attendance (COPD:16%; HF:18%). A further 5% of COPD patients and 4% of HF patients were admitted for an emergency inpatient stay, but not through the ED. A small percentage of patients (COPD:<1%, HF:2%) died without any known contact with secondary care. ED conversion rates at first attendance for both COPD and HF were high: 75% and 79% respectively. A quarter of patients who were not admitted during this first ED attendance attended the ED again within the 30-day follow-up period, and around half (COPD:56%; HF:63%) of these were admitted at this point.

Patients who live alone, had an index admission which included an overnight stay and were comorbid had higher odds of being admitted through the ED than via other routes.

**Conclusion:**

While the majority of patients did not have unscheduled contact with secondary care in the 30 days after index discharge, many patients attended the ED, often multiple times, and many were admitted to hospital, not always via the ED. More frail patients were more likely to be admitted through the ED, suggesting a possible area of focus as discharge bundles are developed.

## Introduction

Hospital readmissions are a longstanding challenge for health providers, as they are relatively common and associated with high financial costs and poor outcomes.[1] Estimates of the proportion of readmissions that are preventable vary, but there is a widely accepted view that many can be avoided.[2] Early readmissions, commonly defined as within 30 days, have become an established indicator of quality of care, and in England and the United States (US), pay for performance initiatives have been introduced to incentivise lowering readmission rates using this time window.[3] By focussing on this one aspect, clinicians and administrators are encouraged to focus on this headline figure without necessarily understanding the complex patient journeys which make up a readmission.[4] For example readmissions ignore other unscheduled return-to-care, specifically emergency department attendances which do not end in admission. A study of a single hospital in the US found that by excluding ED attendances 50% of all return-to-care is ignored and suggested that ‘this may impact on efforts to identify opportunities to improve care transitions’.[5]

Few previous studies have distinguished between different routes to readmission for patients recently discharged from hospital. Across England 71% of all emergency admissions are through the ED, and this proportion is increasing.[6] A similar picture is seen in the US with approximately two-thirds of emergency admissions coming through EDs in 2009, following a steady rise from 2002.[7] It is not known if this is the same for emergency admissions which are within 30 days of discharge from a previous inpatient stay, i.e. readmissions. Better knowledge of these routes and of the kinds of patients that undertake each one is essential for formulating successful improvement efforts.

Heart failure (HF) and chronic obstructive pulmonary disorder (COPD) are major causes of morbidity, poor quality of life, premature death and utilisation of health services.[8, 9] Effective management and treatment of these conditions should reduce the need for emergency admissions which remain frequent and are estimated to cost the National Health Service (NHS) in England over £250 million a year.[10] Given the high rate of readmission for both diseases, often with multiple admissions in one year,[11] a substantial part of this cost can be attributed to readmissions. There is evidence that post-discharge medical care is key to an improved prognosis.[12] For HF, National Institute of Clinical Excellence (NICE) guidelines recommend “a follow-up clinical assessment should be undertaken by a member of the specialist HF team within two weeks of the person being discharged from hospital.”[13] Similar guidelines exist in the US from the American Heart Association. In Britain and Denmark, guidelines recommend that pulmonary rehabilitation start within 4 weeks.[14, 15]

Although much has been published about emergency admissions and readmissions for patients with chronic diseases much less is known about ED attendances following admissions and the pathways to readmissions.

To develop our understanding of patients’ journeys which end in readmission we focussed on patients who had been recently admitted as an emergency for HF or COPD and aimed to determine:

- What proportion of 30-day readmissions are through the ED?
- Does the route of readmission vary by patient characteristics?
- Do patients not admitted for an inpatient stay on their first ED attendance after discharge, re-attend the ED within the 30-day window?
- What is the time interval between these successive visits?

## Methods

### Ethics

We have approval from the Secretary of State and the Health Research Authority under Regulation 5 of the Health Service (Control of Patient Information) Regulations 2002 to hold confidential data and analyse them for research purposes (CAG ref 15/CAG/0005). We have approval to use them for research and measuring quality of delivery of healthcare, from the London—South East Ethics Committee (REC ref 15/LO/0824).

### Data

England’s national hospital administrative database, Hospital Episodes Statistics (HES), comprises all inpatient, day case, outpatient department (OPD) appointment and ED attendance records for all NHS (public) hospitals in England.[16] Records belonging to the same person were linked using a combination of the patient’s unique NHS number, date of birth, sex and postcode; those with an invalid postcode were excluded (<1%). Inter-hospital transfers were linked together to form admissions (“superspells”) and avoid double-counting. The day of final discharge after any inter-hospital transfer was taken as day zero for time-to-event calculation.

We identified a set of adult patients who were discharged alive from a first admission (index admission) with a primary diagnosis of HF (ICD-10 150) and separately COPD (ICD-10 J40-44) that ended between April 2009 and March 2011. We wanted to consider patients for whom this was the first admission so we tracked back 10 years and excluded patients with an admission for HF or COPD during that time. This simplifies the readmission trajectory, as multiple admissions are common in this patient group, and represents an important milestone in the progression of the patient’s disease.[11, 17]

### Outcomes

**Fig 1:**
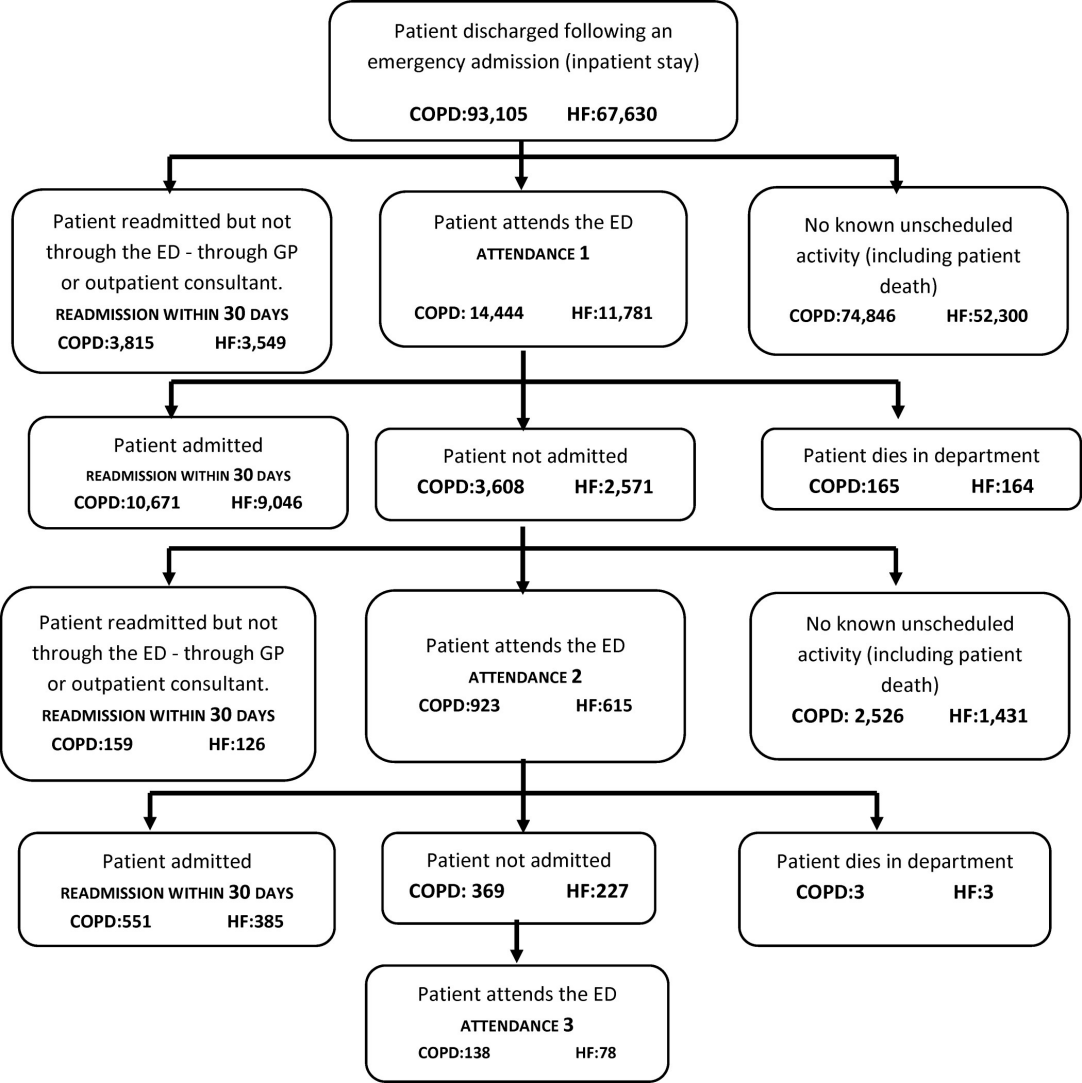
Possible patient routes after discharge from and emergency inpatient stay included in this analysis. Numbers of patients at each stage are shown for each disease. Outcomes within 30 days of the initial discharge were identified.

For all patients discharged alive, patients were followed within the database for 30 days after discharge to determine if they were readmitted as an emergency or attended the ED for any cause (all-cause). Elective admissions and deaths were also identified, again all-cause. All patients who attended the ED but were not admitted continued to be tracked and the first activity following the ED attendance was identified. This process was repeated for patients who were not admitted at the ED a second time. For all patients, we stopped tracking patients 30 days after the discharge from their first (index) emergency admission. Possible routes to readmission are summarised in Fig 1. 30 days was selected as it is the time period used for pay for performance initiatives in England.[18] Patients who have an elective admission following the index admission may have had unscheduled contact following their elective admission. However, we did not include these in further analysis, even if they fell within the 30-day window, as it is not possible to determine which admission the unscheduled contact may be connected with. This is standard when calculating readmission measures, as the intention is to associate the readmission with the admission immediate before it in time.

### Analysis

The majority of analysis was descriptive, identifying proportions of patients with different activities following discharge from an emergency admission. In order to determine associations between patient and hospital stay characteristics and the route to readmission we utilised logistic regression to predict a dichotomous variable ‘readmitted through the ED’ or ‘other route to readmission’. After unadjusted testing of characteristics that were considered to be potentially associated with route to readmission, multivariable logistic models were used to determine the associations of patient characteristics with route of admission. As we were primarily interested in all potential associations between our candidate variables and route to readmission we retained all variables in the model (these are shown in the results section). Results were reported as odds ratio (OR) with 95% confidence intervals (CI). To account for the highly skewed nature of length of stay we categorised length of stay. The relationship between age and route to readmission was investigated using graphical approaches and found not to be linear; for this reason age was also categorised. We assessed logistic regression model performance with the goodness-of-fit test and plots of Hosmer and Lemeshow. Classification performance was assessed using the c-statistic, equivalent to the area under the curve (AUC) for the receiver operator characteristic (ROC) curve.

All analysis was carried out using SAS v9.4.

## Results

### Patient cohort

After excluding patients who had had an admission for COPD or HF in the previous 10 years, between April 2009 and March 2011, 93,105 COPD patients and 67,630 HF were discharged alive following an emergency admission for COPD and HF respectively. Table 1 summarises the characteristics of patients discharged from hospital. The majority of patients were aged over 65 and had one or more comorbidities. Over three-quarters of HF patients spent three or more nights in hospital during their index admission; just over 60% of COPD patients had equivalent stays. One in ten COPD patients were admitted and discharged on the same day, nearly twice as common as for HF patients.

**Table 1:** Summary of discharged patients and the proportion of each group making emergency contact with secondary care or being admitted within 30 days.

		Number (%) of patients discharged alive	
		COPD	HF
**Totals**		93,105	67,630
***Factors relating to the patient or the index admission ***			
**Gender**			
	**Male**	44,888 (48.2%)	33,932 (50.2%)
	**Female**	48,217 (51.8%)	33,698 (49.8%)
**Age stratified by gender**			
**Under 65**	**Male**	11,613 (12.5%)	5,424 (8.0%)
	**Female**	13,528 (14.5%)	2,692 (4.0%)
**65–79**	**Male**	21,065 (22.6%)	13,799 (20.4%)
	**Female**	20,697 (22.2%)	10,317 (15.3%)
**80+**	**Male**	12,210 (13.1%)	14,709 (21.8%)
	**Female**	13,992 (15.0%)	20,689 (30.6%)
**No. of comorbidities**			
	**0**	24,790 (26.6%)	2,886 (4.3%)
	**1–4**	56,629 (60.8%)	44,076 (65.2%)
	**4+**	11,686 (12.6%)	20,668 (30.6%)
**Living alone**			
	**Yes**	9,659 (10.4%)	7,944 (11.8%)
	**No**	83,446 (89.6%)	59,686 (88.3%)
**Index LOS**^**1**^ **(number of nights)**			
	**Zero**	10,251 (11.0%)	3,987 (5.9%)
	**1 or 2**	23,291 (25.7%)	10,432 (15.4%)
	**3+**	58,933 (63.3%)	53,211 (78.7%)
***Factors relating to period after discharge*:**			
**Any OPD**^**2**^ **appt within 14 days**			
	**Yes**	8,863 (9.5%)	18,925 (28.0%)
	**No**	84,242 (90.5%)	48,705 (72.0%)

^1^ LOS: Length of stay

^2^ OPD: Outpatient department

Scheduled Secondary Care Contacts

The first contact for a small number of patients was an elective admission (3042 COPD patients (3% of those discharged alive) and 2874 HF patients (4%)). This could be any elective procedure, as an inpatient or day case. As described in the method these patients were not included in further analysis.

### First unscheduled secondary care contact

Table 2 shows the first contact with secondary care within 30 days of discharge from their index admission (excluding elective admission described above). The majority of patients had no known unscheduled contact with secondary care within 30 days. 21% of COPD and 25% of HF patients had at least one unscheduled contact. The most common contact was an ED attendance (COPD: 16%; HF:18%). The majority of attendances resulted in admission (COPD:74.5%; HF: 78.5%). 4% of COPD patients and 5% of HF patients were readmitted for an emergency inpatient stay via a route other than the ED. For over 50% of patients the first unscheduled activity was over a week from discharge, but less than 14 days (median numbers of days to first activity are shown in Table 3).

**Table 2:** First unscheduled activity after the index inpatient discharge (elective admissions have been included for additional information, although these are not ‘unscheduled’). All outcomes are all-cause.

First unscheduled activity after index inpatient discharge	COPD	Heart Failure
**No known unscheduled contact**	73,641 (79%)	50,455 (75%)
**Died without unscheduled contact secondary care (or an elective admission).**	1,205 (1%)	1,575 (2%)
**Attended the ED but was not admitted as an inpatient**	3,773 (4%)	2,735 (4%)
**Attended the ED and was admitted as an inpatient**	10,671 (12%)	9,046 (14%)
**Admitted as an inpatient–not through the ED.**	3,815 (4%)	3,549 (5%)
		
**ED conversion rate (proportion admitted of those attending the ED)**	74.5%	78.5%

**Table 3:** Time to first unscheduled event and first event after not being admitted following an ED attendance.

	Median (IQR) number of days to FIRST event within 30 days		Median (IQR) number of days to SECOND event–patients not admitted at ED during their first visit.	
Activity	COPD	HF	COPD	HF
**Patient died without unscheduled contact with secondary care.**	10 (4 to 19)	9 (4 to 17)	2 (1 to 5)	5 (1 to 12)
**Patient attended ED, whether admitted at that visit or not**	9 (4 to 17)	10 (4 to 18)	5 (2 to 10)	4 (2 to 9)
**Patient was readmitted as an emergency but not through ED**	10 (5 to 18)	10 (4 to 18)	2 (1 to 6)	2 (1 to 9)
**Patient admitted as an elective admission**	14 (7 to 21)	15 (8 to 22)	9.5 (5 to 16)	9 (3 to 16)

### Route to readmission

For patients who were admitted on their first unscheduled contact with secondary care, the majority (73% for COPD and 72% for HF) came through the ED, but at least one in four patients were admitted through other routes, e.g., through an outpatient clinic or direct admission in association with a primary care physician.

Results of multivariable logistic regression, with routes to readmission modelled as a binary variable (through the ED or other), show that there are significant differences in the characteristics of patients readmitted by different routes (Table 4). COPD patients living alone were 29% more likely to be admitted via the ED; the effect was slightly lower for HF patients (24%). An increasing number of comorbidities was associated with higher odds of being admitted via the ED for HF patients. A similar but not significant pattern was seen for COPD patients. Index admissions of stays of one or more nights were associated with higher odds of being admitted via the ED. Patients who had any OPD appointment in the first two weeks following discharge were less likely to be admitted through the ED. Readmission for the index condition was only significantly associated with route of admission for HF patients, for whom readmission for HF was associated with 18% lower odds of being readmitted via the ED.

**Table 4:** Factors associated with route of readmission. Odds ratios (ORs) presented are the odds of being admitted through the ED compared to ‘other routes’.

	COPD			HF		
	N (%)^a^	Unadjusted ORs (95% CI)	Adjusted ORs (95% CI)	N (%)^a^	Unadjusted ORs (95% CI)	Adjusted ORs (95% CI)
** All admitted patients**	14486			12,595		
**Factors relating to the patient or the index admission **						
**Age**						
**<65**	2,889 (19.9%)	1	1	1,332 (10.6%)	1	1
**65–79**	6,493 (44.8%)	0.96 (0.87, 1.06)	0.92 (0.83, 1.02)	4,401 (34.9%)	1.07 (0.94, 1.22)	1.02 (0.90, 1.17)
**85+**	5,104 (35.2%)	1.04 (0.94, 1.15)	0.97 (0.87, 1.08)	6,862 (54.5%)	1.21 (1.06, 1.37)**	1.10 (0.96, 1.25)
**Sex**						
**Male**	7,398 (51.1%)	1	1	6,374 (50.6%)	1	1
**Female**	7,088 (48.9%)	1.03 (0.95, 1.11)	1.02 (0.94, 1.09)	6,221 (49.4%)	1.07 (0.99, 1.16)	1.04 (0.96, 1.22)
**No. of comorbidities**						
**0**	2,675 (18.5%)	1	1	453 (3.6%)	1	1
**1–3**	8,961 (61.9%)	1.05 (0.95, 1.16)	1.05 (0.95, 1.16)	7,247 (59.0%)	1.30 (1.06, 1.59)**	1.24 (1.01, 1.51)*
**4+**	2,850 (19.7%)	1.13 (1.00, 1.27)	1.12 (0.99, 1.27)	4,715 (37.4%)	1.45 (1.18, 1.78)**	1.37 (1.11, 1.69)**
**Living Alone**						
**No**	12,695 (87.6%)	1	1	10,972 (87.1%)	1	1
**Yes**	1,791 (12.4%)	1.36 (1.20, 1.53)***	1.29 (1.15, 1.46)***	1,623 (12.1%)	1.31 (1.16, 1.48)***	1.24 (1.10, 1.40)***
**Index LOS** **(number of nights)**						
**0**	1,413 (9.8%)	1	1	770 (6.1%)	1	1
**1–2**	3,151 (21.8%)	1.24 (1.08, 1.42)***	1.23 (1.07, 1.41)***	1,841 (14.6%)	1.33 (1.11, 1.59)***	1.30 (1.08, 1.55)***
**3+**	9,922 (68.5%)	1.31 (1.16, 1.48)***	1.31 (1.15, 1.48)***	9,984 (7.3%)	1.46 (1.25, 1.70)***	1.39 (1.19, 1.63)***
**Factors relating to period after discharge:**						
**Any OPD appt within 14 days of index discharge**						
**No**	12,824 (88.5%)	1	1	8,820 (70.0%)	1	1
**Yes**	1,662 (11.5%)	0.68 (0.61, 0.76)***	0.70 (0.63, 0.79)***	3,775 (30.0%)	0.81 (0.75, 0.88)***	0.83 (0.76, 0.90)***
**Factors related to the admission**						
**Readmission LOS (number of nights)**						
0	1,326 (9.2%)	1	1	1,077 (8.6%)	1	1
1–2	2,802 (19.4%)	1.31 (1.13, 1.51)**	1.28 (1.10,1.48)**	2,194 (17.5%)	0.96 (0.83, 1.10)	1.06 (0.90, 1.25)
3+	10,292 (71.4%)	1.19 (1.05, 1.35)**	1.13 (1.00, 1.29)**	9,273 (73.9%)	1.09 (0.92, 1.28)	0.94 (0.82, 1.08)
**Readmission was for index condition**						
No	8,764 (60.4%)	1	1	8,946 (71.0%)	1	1
Yes	5,740 (39.6%)	1.00 (0.93, 1.08)	1.02 (0.95, 1.10)	3,649 (29.0%)	0.79 (0.73, 0.86)***	0.82 (0.76, 0.90)***

^a^The total number of patients admitted and percentage of those admitted.

*p<0.05

**p<0.01

***p<0.001

The c-statistic for both models was low (COPD: 0.55; HF 0.56) suggesting poor discrimination. The Hosmer and Lemeshow plots showed some minor miscalibration at the extremes of risk but model fit was acceptable.

Patients who were not admitted and discharged from ED alive at their first ED visit were followed up in the database for 30 days post-discharge from their index (original) admission. A small number of patients died during their first ED attendance (for COPD, 1.1% of those attending ED as their first activity, 1.4% for HF patients) or were admitted on the same day as their ED attendance without a second attendance: these were excluded from further analysis (Table A and Figures A and B in S1 Supplementary Materials).

We now consider the second and third ED attendances following the index inpatient discharge.

### Second and third ED attendances

25.6% of COPD patients and 27.5% of HF patients re-attended ED after being discharged alive from their first ED attendance without admission for an inpatient stay. Over half of these did so within five days of their first attendance at ED (Table 3). Conversion rates were lower than for their first ED attendance, although still over 50% for both groups of patients. For patients not admitted during the second attendance, 39% of COPD patients and 36% of HF patients attended a third time within the 30 days. At the third attendance, conversion rates dropped to 41% for COPD and 43% for HF.

Of the patients discharged alive from their first ED attendance, 25.0% of COPD patients and 21.5% of HF patients were subsequently admitted within the 30 days, approximately three-quarters of these via ED. Of those admitted via other routes, this happened in two days or less for over 50% of patients (Table 3).

For patients not admitted during the second attendance, 37.5% of COPD patients and 33.8% of HF patients attended a third time within the 30 days. On the third attendance conversion rates dropped to 42.1% for COPD and 42.7% for HF.

## Discussion

### Main findings

The majority of patients (79% for COPD and 75% for HF) had no unscheduled contact with health care within 30 days of discharge. The most common unscheduled contact was ED attendance resulting in an admission (COPD: 12% and HF: 14%), most commonly within 10 days. ED conversion rates at first attendance for both COPD and HF were high: 75% and 79% respectively. Admission via ED was the most common route (73.9% for COPD and 71.8% for HF), and these patients differed from those admitted via other routes. Patients with more comorbidities and who live alone were more likely to be readmitted through the ED. Patients for whom their index admission did not include an overnight stay had over 20% higher odds of being admitted though the ED. In contrast, patients who had an OPD appointment within 14 days of discharge had lower odds of being admitted through the ED (30% lower for COPD and 17% lower for HF patients).

A quarter of patients who were not admitted on their first ED attendance attended the ED again within the 30-day post-discharge period, usually in less than one week, and over half of these were admitted. The conversion (admission) rate for the second attendance was lower than the first attendance and fell again for the third visit.

Patterns for COPD and HF were similar.

To date most previous studies of patients with chronic diseases who were recently discharged from their inpatient stay focus on readmissions, though few considered the route of admission. Patients who attend the ED after index inpatient discharge but are sent home at that visit and then re-attend the ED have been particularly little studied. In our study we found that most patients had no further unscheduled contact with hospitals within 30 days of discharge, although readmissions were common. These readmission figures are similar to those in the US.[1] Studies in England and the US show that a high proportion of all emergency admissions were through the ED, reflecting the models of unscheduled care. We found a lower percentage of patients attended the ED than a single-site study in the US,[5] although this may reflect different routes to readmission. The high ED conversion rates mean that excluding ED attendances which do not end in admission excludes approximately one quarter of unscheduled contacts, lower than the 50% found by Rising et al.[5] This may be due to the severity of the diseases we studied, though admission thresholds, which are influenced by bed availability and other factors, may also explain the difference.

Few studies have looked at associations between patient characteristics and admission route. A descriptive analysis of English admissions suggested that older patients and patients from ethnic minorities were more likely to be admitted via the ED;[18] similar results were seen by Kocher et al in the US.[19] A single-state study in the US found that elderly and patients with comorbidities were more likely to be admitted through the ED and married patients were less likely to be admitted via this route. In line with the results of the US study, we found that patients with multiple comorbidities and patients who live alone were all more likely to be readmitted via the ED than other routes, suggesting similar patterns for readmissions as for all emergency admissions. However, we did not find that older patients were more likely to be admitted via the ED. In addition, we considered the length of stay of the index admission, and found that index admissions which included at least one overnight stay were more likely to be admitted via the ED. These longer index stays may be a proxy for severity and/or frailty. We suggest that for patients recently discharged from an emergency admission ‘frail’ patients were more likely to be admitted through the ED. This is important as it may indicate lack of access to either primary or specialist care for certain groups is resulting in higher use of ED. An association between access and ED use has been shown in primary care [20, 21] but not specialist care. Bottle et al have shown that for heart failure patients, access to cardiology outpatient appointments within the recommended 14 days varies by age, with older patients less likely to have had access to specialist follow-up care,[11] but not that this was connected to ED use. In addition, the context of the ED may be an important factor, as non-medical factors such as patients’ living arrangements, can affect the admission threshold.[22]

For patients who had unscheduled contact, the time between attendances and readmissions was relatively short, less than two weeks. This is in keeping with Dharmarajan et al, who found that the daily risk of readmission was almost twice as high on day seven than on day 30.[23]

We found that a small proportion of patients returned to the ED after being discharged without admission. It is easy to characterise these patients as being a burden on health services. However, they can also be conceptualised as having a significant ‘personal burden’, experiencing ongoing symptoms and anxieties which have not been yet been eased.[24]

### Limitations

This is a large study which considers all patients who were discharged following an emergency admission for COPD or HF in England over a two-year period.

We focussed on the first 30 days following discharge, though it is known that the period of activity following an emergency admission is not limited to 30 days.[17] This time limit means that we did not capture all patients who returned to the ED after discharge without admission, as for some patients this will fall outside of the 30-day limit. This also means that the number of days to second ED visit will be shorter as it is capped to fall within the specified time period, which will be less than 30 days. However, the 30-day window is the dominant one for studying and monitoring readmissions and will contain a higher proportion of contacts related to the index admission than longer follow-up times.

The poor model performance in terms of discrimination and goodness of fit suggest that there may be unmeasured confounders which are not included in the model and would improve model discrimination and fit.

This analysis focuses on contacts and readmission routes. We have published elsewhere patient factors associated with increased risk of readmission and ED attendance.[25]

There may have been changes in practices and policies since the study period. However, we believe this analysis provides a useful approach to analysing readmissions and provides an important baseline to determine the impact of interventions and pressures faced by hospitals.

To deepen our understanding of patient contacts after discharge, it would be useful to have information on severity as well as contacts with primary and community care after discharge, but information such as breathlessness scales or ejection fraction were not captured by the database.

## Conclusions

Whilst the majority of patients did not have unscheduled contact with secondary care after discharge, there was a high number of unscheduled health care contacts with associated unplanned costs. Some did so multiple times within the 30-day window. We found some notable differences in patient characteristics by route to readmission, with more frail patients more likely to be admitted through the ED, suggesting a need for a more integrated approach for this group of patients. It is key that these unscheduled contacts are reduced, to reduce both the burden on the NHS and ‘personal burden’ experienced by the patient.

## Supporting information

S1 Supplementary Materials Detailed information on outcomes of first ED visits post-discharge. **Table A** Outcome of first ED visit post discharge.
**Figure A** Unscheduled activity for patients who attended ED but were not admitted (COPD).
**Figure B** Unscheduled activity for patients who attended ED but were not admitted (HF).
(DOCX)

## References

[1] Jencks SF, Williams MV, Coleman EA. Rehospitalizations among patients in the Medicare fee-for-service program. The New England journal of medicine. 2009;360. 10.1056/NEJMsa0803563 19339721

[2] van Walraven C, Jennings A, Forster AJ. A meta-analysis of hospital 30-day avoidable readmission rates. J Eval Clin Pract. 2012;18(6):1211–1218. 10.1111/j.1365-2753.2011.01773.x 22070191

[3] Balicer RD, Shadmi E, Israeli A. Interventions for reducing readmissions—are we barking up the right tree? Isr J Health Policy Res. 2013;2(1):2 10.1186/2045-4015-2-2 23343051 PMC3570430

[4] Joynt KE, Jha AK. Thirty-Day Readmissions—Truth and Consequences. New England Journal of Medicine. 2012;366(15):1366–1369. 10.1056/NEJMp1201598 22455752

[5] Rising K, White L, Fernandez W, Boutwell A. Emergency department visits after hospital discharge: a missing part of the equation. Annals of Emergency Medicine. 2013;62(2). 10.1016/j.annemergmed.2013.01.024 23562776

[6] Cowling TE, Soljak MA, Bell D, Majeed A. Emergency hospital admissions via accident and emergency departments in England: time trend, conceptual framework and policy implications. J R Soc Med. 2014;107(11):432–438. 10.1177/0141076814542669 25377736 PMC4224646

[7] Morganti KG, Bauhoff S, Blanchard JC, Abir M, Iyer N, Smith A, et al The Evolving Role of Emergency Departments in the United States. Rand Health Q. 2013;3(2):3 28083290 10.7249/rhq-03-02-03 PMC4945168

[8] Inamdar AA, Inamdar AC. Heart Failure: Diagnosis, Management and Utilization. Journal of Clinical Medicine. 2016;5(7):62. 10.3390/jcm5070062 PMC4961993 27367736

[9] Guarascio AJ, Ray SM, Finch CK, Self TH. The clinical and economic burden of chronic obstructive pulmonary disease in the USA. Clinicoecon Outcome Res. 2013;5:235–245. 10.2147/CEOR.S34321 PMC3694800 23818799

[10] Trueman D, Woodcock F, Hancock E. Estimating the economic burden of respiratory illness in the UK British Lung Foundation;2017.

[11] Bottle A, Goudie R, Bell D, Aylin P, Cowie MR. Use of hospital services by age and comorbidity after an index heart failure admission in England: an observational study. BMJ Open. 2016;6(6). 10.1136/bmjopen-2015-010669 PMC4908910 27288372

[12] Laverty AA, Elkin SL, Watt HC, Millett C, Restrick LJ, Williams S, et al Impact of a COPD Discharge Care Bundle on Readmissions following Admission with Acute Exacerbation: Interrupted Time Series Analysis. PLoS ONE. 2015;10(2):e0116187 10.1371/journal.pone.0116187 25679218 PMC4332682

[13] NICE. Chronic heart failure in adults: management. Clinical guideline [CG108]. 2010.

[14] Bolton CE, Bevan-Smith EF, Blakey JD, Crowe P, Elkin SL, Garrod R, et al British Thoracic Society guideline on pulmonary rehabilitation in adults: accredited by NICE. Thorax. 2013;68(Suppl 2):ii1–ii30. 23880483 10.1136/thoraxjnl-2013-203808

[15] Morsø L, Jensen MS, von Plessen C, Qvist P. Rehabilitation of Discharged Patients With Chronic Obstructive Pulmonary Disease—Are New Strategies Needed? Health Services Research and Managerial Epidemiology. 2017;4:2333392816687704 10.1177/2333392816687704 28508012 PMC5415270

[16] NHS Hospital Episode Statistics (HES) Available online: https://digital.nhs.uk/data-and-information/data-tools-and-services/data-services/hospital-episode-statistics Accesed[July 13 2018]

[17] Bottle A, Aylin P, Bell D. Effect of the readmission primary diagnosis and time interval in heart failure patients: analysis of English administrative data. European journal of heart failure. 2014;16(8):846–853. 10.1002/ejhf.129 25044392

[18] NHS England Pricing Team Guidance for commissioners on the marginal rate emergency rule and 30-day readmission rule. 2016 NHS England Publication Gateway reference: 05995

[19] Kocher KE, Nallamothu BK, Birkmeyer JD, Dimick JB. Emergency Department Visits After Surgery Are Common For Medicare Patients, Suggesting Opportunities To Improve Care. Health Affairs. 2013;32(9):1600–1607. 10.1377/hlthaff.2013.0067 24019365

[20] Cowling TE, Harris M, Watt H, Sojak M, Richards E, Gunning E, et al Access to primary care and the route of emergency admission to hospital: retrospective analysis of national hospital administrative data. BMJ Quality & Safety. 2016;25(6):432–440. 10.1136/bmjqs-2015-004338 PMC4893129 26306608

[21] Barker I, Steventon A, Deeny SR. Association between continuity of care in general practice and hospital admissions for ambulatory care sensitive conditions: cross sectional study of routinely collected, person level data. BMJ. 2017;356. 10.1136/bmj.j84 28148478

[22] Lewis EF, Hardy M, Snaith B. Estimating the effect of nonresponse bias in a survey of hospital organizations. Eval Health Prof. 2013;36. 10.1177/0163278713496565 23908382

[23] Dharmarajan K, Hsieh AF, Kulkarni VT, Lin Z, Ross JS, Horwitz L, et al Trajectories of risk after hospitalization for heart failure, acute myocardial infarction, or pneumonia: retrospective cohort study. BMJ. 2015;350:h411 10.1136/bmj.h411 25656852 PMC4353309

[24] Daniels J, Osborn M, Davis C. Better safe than sorry? Frequent attendance in a hospital emergency department: an exploratory study. British Journal of Pain. 2018;12(1):10–19. 10.1177/2049463717720635 29416860 PMC5788111

[25] Bottle A, Honeyford K, Chowdhury F, Bell D, Aylin P. What are the determinants of variations in emergency readmission rates and one-year mortality in patients hospitalized with heart failure or chronic obstructive pulmonary disease? Observational study using national administrative data. Health Services and Delivery Research. Southampton (UK): NIHR Journals Library; 2018 Jul. (Health Services and Delivery Research, No. 6.26.) 10.3310/hsdr062602018 In Press.

