# Supplementary material for: Unscheduled hospital contacts after inpatient discharge: A national observational study of COPD and heart failure patients in England
Source: PLoS One. 2019 Jun 13;14(6):e0218128. doi: 10.1371/journal.pone.0218128 (PMC6563993; doi:10.1371/journal.pone.0218128)
Supplement: S1 Supplementary Materials — Table A Outcome of first ED visit post discharge. Figure A Unscheduled activity for patients who attended ED but were not admitted (COPD). Figure B Unscheduled activity for patients who attended ED but were not admitted (HF). (DOCX) [file pone.0218128.s001.docx]

Supplementary Materials – Detailed information on outcomes of first ED visits post-discharge

Table A shows a detailed breakdown of the outcome of patients who visit ED after discharge from an emergency admission. Just over 1% of patients who attend ED but are not admitted die in ED. After excluding patients who make a second visit to ED on the same day, 2.5% of COPD patients and 2.8% of HF patients who attend ED and are not coded as admitted are coded as being admitted to a hospital as an emergency that day. It is not possible to determine if they are admitted through ED and there is an error in ED data or if these patients, having been discharged from ED, are subsequently admitted through a different route.

| **Activity** | **COPD** | **HF** |
| --- | --- | --- |
| **Patient admitted** | 10671 (73.9%) | 9046 (76.8%) |
| **Patient died on day of visit without an admission** | 165 (1.1%) | 164 (1.4%) |
| **Patient had an emergency admission recorded on that day, but not coded as an ED admission** | 356 (2.5%) | 332 (2.8%) |
| **Not admitted** | 3340 (22.5%) | 2288 (19.0%) |
| **Total number of patients** | 14444 | 11781 |

Table A. Outcome of first ED visit post discharge.

**COPD**

ED conversion (admission) rate:

55.6%

Figure A. Unscheduled activity for patients who attended ED but were not admitted (COPD).

ED conversion (admission) rate:

62.6%

**HF**

Figure B. Unscheduled activity for patients who attended ED but were not admitted (HF).
